# A Laboratory Methodology for Dual RNA-Sequencing of Bacteria and their Host Cells *In Vitro*

**DOI:** 10.3389/fmicb.2017.01830

**Published:** 2017-09-21

**Authors:** James W. Marsh, Michael S. Humphrys, Garry S. A. Myers

**Affiliations:** ^1^School of Life Sciences, The ithree institute, University of Technology Sydney Ultimo, NSW, Australia; ^2^Institute for Genome Sciences, University of Maryland School of Medicine Baltimore, MD, United States

**Keywords:** dual RNA-Seq, bacteria, host, *Chlamydia*, protocol

## Abstract

Dual RNA-Sequencing leverages established next-generation sequencing (NGS)-enabled RNA-Seq approaches to measure genome-wide transcriptional changes of both an infecting bacteria and host cells. By simultaneously investigating both organisms from the same biological sample, dual RNA-Seq can provide unique insight into bacterial infection processes and reciprocal host responses at once. However, the difficulties involved in handling both prokaryotic and eukaryotic material require distinct, optimized procedures. We previously developed and applied dual RNA-Seq to measure prokaryotic and eukaryotic expression profiles of human cells infected with bacteria, using *in vitro Chlamydia*-infected epithelial cells as proof of principle. Here we provide a detailed laboratory protocol for *in vitro* dual RNA-Seq that is readily adaptable to any host-bacteria system of interest.

## Introduction

The complex interplay of infecting bacteria with their eukaryotic host is key to understanding pathogenesis and disease progression (Humphrys et al., 2013). Dual RNA-Seq is a powerful method to investigate these infection dynamics. By simultaneously capturing expressed genes of both the pathogen and the host, dual RNA-Seq can summarize the molecular interplay of bacterial infection processes and the reciprocal host responses. We previously developed and applied dual RNA-Seq to *Chlamydia*-infected eukaryotic cells as proof-of-principle (Humphrys et al., 2013). Dual RNA-Seq has been also successfully applied to several other bacteria-host pairings (Camilios-Neto et al., 2014; Avican et al., 2015; Baddal et al., 2015; Brogaard et al., 2015; Rienksma et al., 2015; Aprianto et al., 2016; Westermann et al., 2016).

RNA-Seq has several benefits over other transcriptional profiling methods. Microarrays suffer from high background noise, cross hybridization issues, and are typically restricted to known or predicted mRNAs (Lister et al., 2008; Shendure, 2008; Wang et al., 2009; Oosthuizen et al., 2011). The large size of eukaryotic genomes typically excludes the use of tiling arrays to measure antisense RNA expression and other non-coding RNA transcripts; conversely, tag-based sequencing suffers from partial coverage of transcripts by design (Bertone et al., 2004). These drawbacks are largely alleviated by RNA-Seq-based methods, which enable genome-scale coverage, the measurement of absolute expression levels, and minimal background signal (Marioni et al., 2008; Fu et al., 2009; Kawahara et al., 2012). Furthermore, RNA-Seq can detect transcribed intronic and intergenic regions, as well as post-transcriptional regulatory events such as alternative splicing and differential isoform expression (Mortazavi et al., 2008; Kalam et al., 2017).

However, sequencing mixed prokaryotic and eukaryotic populations presents several challenges. For example, bacterial RNAs can constitute <1% of the total RNA in an infected cell. Moreover, up to 98% of total RNA in an infected cell is eukaryotic ribosomal RNA (rRNA), requiring rRNA depletion or mRNA enrichment strategies to ensure sufficient non-rRNA can be sequenced at reasonable cost (Giannoukos et al., 2012). Additionally, traditional cell lysis techniques are often not suitable for both eukaryotic and prokaryotic organisms at once. Finally, the trade-off between the multiplicity of infection (MOI) used and sequencing depth (cost) can lead to a mixed population of infected and uninfected cells, which may bias results.

**Figure 1:**
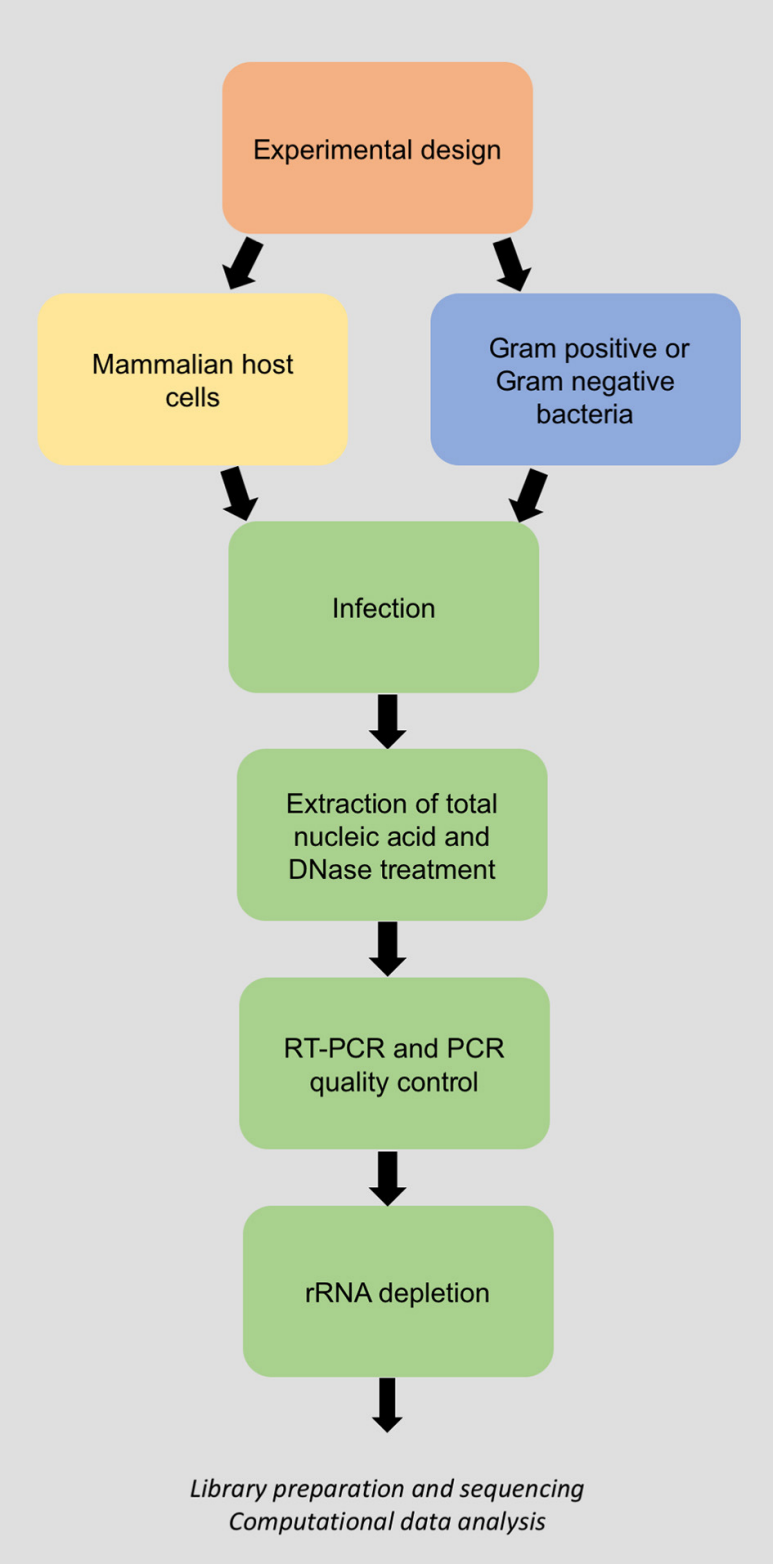
Flow chart of the laboratory methodology for dual RNA-Sequencing of bacteria and their host.

A complete dual RNA-Seq experiment comprises three stages: (1) Total host-bacteria RNA extraction and purification; (2) next-generation sequencing (NGS) of total RNA; and (3) bioinformatic processing and statistical analysis of the host-bacteria transcriptome. Here we present a laboratory methodology for stage (1) of a typical *in vitro* dual RNA-Seq experiment. The steps are described for host cell infection, total host-bacteria RNA extraction and rRNA depletion, RT-PCR quality control, and RNA quantification (Figure 1). This RNA can then be used as input for stages (2) and (3) of the dual RNA-Seq experiment. While we provide some general guidelines and recommendations for NGS and data analysis, detailed pipelines have been described in detail previously (Marsh et al., 2017). The protocol has been optimized according to yield, time, throughput, reproducibility, and quality, and is widely applicable to diverse pairings of mammalian cell line and bacterial species. Wherever possible, we routinely use commercially available kits due to their reliability and reproducibility, however we have carefully optimized the manufacturer's instructions to suit this dual RNA-Seq protocol.

### Experimental design

For dual RNA-Seq, a typical workflow includes RNA extraction, eukaryotic, and prokaryotic rRNA depletion, library preparation, sequencing, and analysis (Figure 1). However, a careful assessment of experimental design is crucial before these steps are attempted. The biological question(s) of interest is the starting point, whether it is a hypothesis-driven process via use of bacterial mutants and/or host cell knockout/knockdown mutants, or a hypothesis discovery experiment that, for example, examines organisms that are not amenable to genetic manipulation. The questions under examination will influence the RNA moieties to be investigated (e.g. mRNA, miRNA, ncRNA etc.), appropriate controls, the MOI, any time points, the total RNA quantity required, and sequencing depth.

The choice of organism, both host and bacteria, is central to the question(s) of interest and will inform much of the experimental design. Host cells should be selected for their biological relevance in relation to the bacterium. The identification of host differentially expressed genes (DEGs) or isoforms relative to mock-infected controls is the standard approach; additional controls should also be considered to differentiate specific from non-specific host responses to the bacterium. The choice of bacterium is dependent on the infection system under study, and can be expanded to compare (host or bacterial) transcriptional differences in the presence of different bacterial species, virulent vs. avirulent strains, or mutant vs. wild-type strains.

The ability of a dual RNA-Seq experiment to accurately determine differential gene expression between conditions is contingent on obtaining minimal biological and technical variation, which can be addressed by managing the trade-off between the number of replicates and sequencing depth (Auer and Doerge, 2010; Yu et al., 2017). We suggest that at least three, but preferably six, biological rather than technical replicates should be included to minimize statistical error and provide more biologically meaningful data; increasing sequencing depth is a secondary priority (Oshlack et al., 2010). While a larger number of replicates can become statistically unwieldy it will also enable a greater amount of variation to be captured, decreasing the rate of Type I errors (false positives). RNA spike-ins and unique molecular identifiers (UMI) can also be included to quantify absolute RNA levels if this is of interest (Jiang et al., 2011; Parekh et al., 2016).

As the transcriptional responses of bacteria and host are likely to occur at different times and to different degrees, any time points should be selected to capture each stage of the infection process. At very early times of infection, there is likely to be a limited quantity of bacterial RNA present, particularly when low MOIs are used. To address this, we typically opt to increase sequencing depth to ensure sufficient bacterial sequence reads are obtained.

It is beneficial to predict the quantity of RNA required during the experimental design stage, and this will be influenced by the number of samples, conditions, replicates, and time-points, as well as the sequencing technology used. It is important to note that total RNA quantity will vary according to both biological (sample type, cellular metabolism) and technical (cell lysis, RNA purification) reasons. Additionally, enough RNA should be available to complete the QC stages of the experiment, including RT-PCR, agarose gel electrophoresis, and Bioanalyzer analysis. At the sequencing stage, the method of library preparation will dictate how much RNA is required, which usually lies within the microgram to picogram range. However, a useful convention is that more RNA input will require less amplification during sequencing, resulting in superior library complexity. As a guide, for mammalian cell culture-based dual RNA-Seq experiments, one well of a six-well plate results in ~100 ng of host RNA and ~500 pg bacterial RNA.

A sequencing depth that addresses the project objectives is essential and it is recommended that ~5 × 10^8^ host reads and >1 × 10^6^ bacterial reads are required for adequate coverage (Toung et al., 2011; Westermann et al., 2012). To achieve this will depend on the organisms under investigation, but here we present the calculations for a *Chlamydia*-infected human host cell as a working example. First the genome size is calculated for each organism; *Chlamydia* has a genome size of 1.04 Mb and the *Homo sapiens* genome size is 3253.9 Mb. The ratio of the *Chlamydia* genome to the human genome is ~1:3,200 Mb, indicating that chlamydial RNA will account for ~0.03% of total host-bacteria RNA. As ~95% of this will be rRNA and tRNA (Westermann et al., 2012), bacteria and host mRNA will account for ~0.0015 and 4.9985% of total RNA, respectively. Given this ratio, 1 × 10^10^ host reads and ~3.33 × 10^9^ bacterial reads are required to capture sufficient RNA from both organisms. Thus, with the majority of bacterial genomes falling within the range of ~1–5 Mb, at least 1 × 10^10^ reads are needed for a successful dual RNA-Seq experiment using bacteria-infected mammalian host cells in order to achieve sufficient coverage.

Prior to sequencing, it should be considered whether single-end (SE) or paired-end (PE) libraries should be prepared and whether reads should be strand-specific. For dual RNA-Seq we prefer paired-end reads where each RNA fragment is sequenced from both sides, as it doubles the number of reads, reduces the rate of alignment ambiguity, and enables the identification of novel splice isoforms (host). Strand-specific libraries are also recommended, as the usually high gene density and presence of overlapping genes on opposite strands seen in bacterial genomes can more accurately be measured, while allowing the identification of potential antisense transcripts. The capture of the entire transcriptome, including coding, non-coding, anti-sense, and intergenic RNAs enables the discovery of complex and global transcriptional events.

### Infection

When infecting host cells, the bacterial MOI is an important consideration—a suitable infection rate should be selected to maximize the transcriptional response from both organisms while minimizing the number of uninfected cells. Here we describe the use of an MOI of ~1.5 which allows for a ~100% infection; if additional measures are required to enrich the selection of infected cells the researcher can consider increasing the MOI, the sequencing depth, or physically separating infected from infected cells by FACS or laser microdissection (Vannucci et al., 2013; Westermann et al., 2016). However, caution is recommended as an increased MOI can lead to a less biologically relevant host response, while sequencing over a certain threshold can promote transcriptional noise or spurious cDNA transcripts (Tarazona et al., 2011).

The method of infection depends on the host cell and this protocol provides guidance for infecting mammalian cell lines. While the methodology is largely consistent for different cells, the type of media and the required supplements will differ and should be selected according to the host cell. Table 1 provides a list of common cell types and their recommended culturing conditions as a general guideline. As cycloheximide (an inhibitor of protein synthesis often used to maximize chlamydial yields *in vitro*), is not used, the host cells are seeded at ~60% confluency at the time of infection to ensure continued viability throughout the time-course of the study. Given the time-sensitive nature of the experiment, it is crucial to synchronize the initial bacterial infection by centrifugation, followed by the removal of dead or non-viable bacterial cells by washing twice with DPBS.

**Table 1:** Mammalian cell lines and culture medium.

**Cell Line**	**Cell Type**	**Species**	**Tissue**	Medium^*^
293	Fibroblast	Human	Embryonic kidney	MEM and 10% FBS
3T6	Fibroblast	Mouse	Embryo	DMEM, 10% FBS
A549	Epithelial	Human	Lung carcinoma	F-12K, 10% FBS
A9	Fibroblast	Mouse	Connective tissue	DMEM, 10% FBS
AtT-20	Epithelial	Mouse	Pituitary tumor	F-10, 15% horse serum, and 2 .5% FBS
BALB/3T3	Fibroblast	Mouse	Embryo	DMEM, 10% FBS
BHK-21	Fibroblast	Hamster	Kidney	GMEM, 10% FBS, or MEM, 10% FBS, and NEAA
BHL-100	Epithelial	Human	Breast	McCoy'5A, 10% FBS
BT	Fibroblast	Bovine	Turbinate cells	MEM, 10% FBS, and NEAA
Caco-2	Epithelial	Human	Colon adeno carcinoma	MEM, 20% FBS, and NEAA
Chang	Epithelial	Human	Liver	BME, 10% calf serum
CHO-K1	Epithelial	Hamster	Ovary	F-12, 10% FBS
Clone 9	Epithelial	Rat	Liver	F-12K, 10% FBS
Clone M-3	Epithelial	Mouse	Melanoma	F-10, 15% horse serum, and 2 .5% FBS
COS-1, COS-3, COS-7	Fibroblast	Monkey	Kidney	DMEM, 10% FBS
CRFK	Epithelial	Cat	Kidney	MEM, 10% FBS, and NEAA
CV-1	Fibroblast	Monkey	Kidney	MEM, 10% FBS
D-17	Epithelial	Dog	Osteosarcoma	MEM, 10% FBS, and NEAA
Daudi	Lymphoblast	Human	Blood from a lymphoma patient	RPMI-1640, 10% FBS
GH1, GH3	Epithelial	Rat	Pituitary tumor	F-10, 15% horse serum, and 2 .5% FBS
H9	Lymphoblast	Human	T-cell lymphoma	RPMI-1640, 20% FBS
HaK	Epithelial	Hamster	Kidney	BME, 10% calf serum
HCT-15	Epithelial	Human	Colorectal adenocarcinoma	RPMI-1640, 10% FBS
HeLa	Epithelial	Human	Cervix carcinoma	MEM, 10% FBS, and NEAA (in suspension, S-MEM)
HEp-2	Epithelial	Human	Larynx carcinoma	MEM, 10% FBS
HL-60	Lymphoblast	Human	Promyeolocytic leukemia	RPMI-1640, 20% FBS
HT-1080	Epithelial	Human	Fibrosarcoma	MEM, 10% HI FBS, and NEAA
HT-29	Epithelial	Human	Colon adenocarcinoma	McCoy's 5A, 10% FBS
HUVEC	Endothelial	Human	Umbilical cord	F-12K, 10% FBS, and 100 μg/mL heparin
I-10	Epithelial	Mouse	Testicular tumor	F-10, 15% horse serum, and 2 .5% FBS
IM-9	Lymphoblast	Human	marrow from Myeloma patient	RPMI-1640, 10% FBS
JEG-2	Epithelial	Human	Choriocarcinoma	MEM, 10% FBS
Jensen	Fibroblast	Rat	Sarcoma	McCoy's 5A, 5% FBS
Jurkat	Lymphoblast	Human	Lymphoma	RPMI-1640, 10% FBS
K-562	Lymphoblast	Human	Myelogenous leukemia	RPMI-1640, 10% FBS
KB	Epithelial	Human	Oral carcinoma	MEM, 10% FBS, and NEAA
KG-1	Myeloblast	Human	Marrow from erythroleukemia patient	IMDM, 20% FBS
L2	Epithelial	Rat	Lung	F-12K, 10%FBS
LLC-WRC 256	Epithelial	Rat	Carcinoma	Medium 199, 5% horse serum
McCoy	Fibroblast	Mouse	Unknown	MEM, 10% FBS
MCF7	Epithelial	Human	Breast adenocarcinoma	MEM, 10% FBS, NEAA, and 10 μg/mL insulin
WI-38	Epithelial	Human	Embryonic lung	BME, 10% FBS
WISH	Epithelial	Human	Amnion	BME, 10% FBS
XC	Epithelial	Rat	Sarcoma	MEM, 10% FBS, and NEAA
Y-1	Epithelial	Mouse	Tumor of adrenal	F-10, 15% horse serum, and 2.5% FBS

* BME, Basal Medium Eagle; DMEM, Dulbecco's Modified Eagle Medium; FBS, Fetal Bovine Serum; GMEM, Glasgow Minimum Essential Medium; IMDM, Iscove's Modified Dulbecco's Medium; MEM, Minimum Essential Medium; NEAA, Non-Essential Amino Acids Solution; TNM-FH, Trichoplusia ni Medium-Formulation Hink (i.e., Grace's Insect Medium, Supplemented). This table has been adapted from the Cell Culture Basics Handbook (ThermoFisher Scientific); for more information see: https://www.thermofisher.com/content/dam/LifeTech/global/promotions/global/images/aai-2015/aai-pdfs/GibcoCellCultureBasicsHandbook.pdf.

### RNA extraction

To ensure high-quality data, dual RNA-Seq typically requires a relatively large amount of input RNA. Additionally, RNA integrity is critical to the success of the protocol, as degraded samples will result in low yields that are insufficient for sequencing (Cheranova et al., 2013). Extreme care must be taken to prevent DNA contamination or RNA degradation, as this will be detrimental to both the library preparation and subsequent analyses. Degradation can be minimized by adhering to the protocol time and temperature requirements, purchasing highly pure, and RNase-free reagents, and using RNA-free equipment and consumables. Always conduct RNA work in a clean environment that is partitioned from non-RNA work.

There are several commercial kits available for performing the RNA extraction (Table 2), but they differ in their effectiveness and suitability for dual RNA-Seq. In our experience, the kits that utilize bead disruption or column-based extraction suffer from reduced yield and poor quality of the total host-bacteria RNA. Instead, we prefer the salt-precipitation protocol of the MasterPure^TM^ Complete DNA and RNA Purification Kit (Epicenter) with optimized steps to be the most effective at extracting quality RNA from several host-bacteria pairings.

**Table 2:** Commercial RNA extraction kits.

**Kit**	**Catalog No**.	**Manufacturer**	**Lysis Method**	**Extraction Method**
RiboPure Bacteria	AM1925	Ambion	Bead cell disruption	Spin column
PureLink RNA Mini Kit	12183020	Life Technologies	Chemical lysis	Spin column
RNeasy Mini Kit	74104	Qiagen	Chemical lysis	Spin column
MasterPure Complete DNA and RNA Purification Kit	MC85200	Epicenter	Chemical lysis	Salt precipitation
UltraClean Microbial RNA Isolation	12224-50	MoBio	Bead cell disruption	Spin column
High Pure RNA Isolation Kit	11828665001	Roche	Chemical lysis	Spin column

The method of bacteria and host lysis is an important consideration and will depend on the infection system being investigated. We find that the enzymatic lysis included in the MasterPure^TM^ kit is most effective for lysing both host and Gram negative bacteria. For Gram positive bacteria, we include an initial sonication step to break open the cell wall prior to extraction. In each case, both the host and bacterial cells are lysed, releasing endogenous RNases so it is critical to minimize the delay between host cell lysis and RNA extraction to avoid degradation. Host cells are lysed, host proteins digested, and total nucleic acid precipitated with isopropanol. One caveat of the MasterPure extraction kit is the residual contamination of genomic DNA, however we have found that two treatments with TURBO DNA-*free*^TM^ DNase (Thermo Fisher) is most effective, compared to phenol:chloroform and precipitation-based methods.

### RT-PCR quality control

We recommend performing three real-time RT-PCR assays for human targets and one endpoint PCR assay for bacterial targets to confirm DNA removal and the presence of RNA. These assays are based on TaqMan® Gene Expression assays (Applied Biosystems) with primer and probe sets targeting beta actin, mitochondria-encoded ATP synthase 6, and eukaryotic 18S rRNA (Humphrys et al., 2013), and include a reverse transcriptase-minus (RT-) test to detect contaminating DNA and a reverse transcriptase-plus (RT+) test for the detection of RNA. We generally base the endpoint PCR on custom-designed primer sets that are specific for the bacterium of interest. Please consult Table 3 for common problems and solutions relating to this stage.

**Table 3:** Troubleshooting.

**Stage**	**Problem**	**Cause**	**Solution**
Nanodrop	A260/A280 ratio is < 1.8	This indicates protein contamination in the sample. The cell lysis and purification steps were not effective	Decrease the amount of starting material. Use fresh proteinase K; ensure the correct concentration. Increase incubation time or concentration to 100 ug/mL if necessary.
Agarose gel electrophoresis	Two large, prominent bands on gel	These are potentially the 18S and 28S eukaryotic rRNA indicating that rRNA-depletion was not effective	
	Low molecular weight smearing	RNA is potentially degraded	Run on Bioanalyzer to confirm.
Bioanalyzer	Two large peaks at ~30 s	Potentially the 18S and 28S eukaryotic rRNA indicating that rRNA-depletion was not effective	
	Well-defined peaks at ~15 s	Potentially the tRNA and miRNA	Columns were not effective to remove small size fragments.
	Low-molecular weight peaks	RNA is potentially degraded	Ensure work is conducted in an RNase-free environment. Avoid freeze-thawing RNA.
	Low, wide peak at ~30 s	Potential DNA carryover	Increase DNase incubation time.
RNA elution	Low RNA yield	If not degraded, RNA extraction may not have been effective.	Do not freeze-thaw RNA. Increase number of cells. Increase lysis incubation time.
		Cell lysis may be incomplete	Repeat incubation in cell lysis buffer if necessary. For systems that include Gram positive bacteria, ensure that the sonication step is performed to complete, with incubation on ice between each pulse.
RNA purification	Loose pellet	Pellet not completely dried. Centrifugation not effective	Dry sample on ice before adding Protein Precipitation Reagent. Repeat centrifugation step.
RT-PCR	Amplification in RT- control	gDNA contamination	Confirm the integrity of DNase used. Increase incubation time. Ensure that the cell lysis procedure was correctly followed.
	Weak amplification product	Degraded reagents	Avoid freeze-thawing template and reagents. Check the expiry of the reagents. Input template may be too concentrated or too dilute.
		Degraded template	
		No cDNA synthesis	Reduce the temperature for the cDNA synthesis step. Annealing temperatur mnay be too high. Increase the extension time and number of cycles.

### rRNA depletion

As rRNA typically constitutes more than 95% of total RNA, rRNA depletion should be considered to maximize the recovery of mRNA and reduce sequencing depth. It is possible to avoid depletion steps by instead sequencing far more deeply and subsequently removing rRNA reads during analysis. However, the sequencing costs of this strategy are substantial, as most sequence reads will align to rDNA genes and be discarded. Further reductions of sequencing costs per base may permit this strategy in the coming years, but it is currently more cost-effective to deplete rRNA prior to sequencing. There are several commercial kits available for nuclease digestion and size-selection; this protocol utilizes both a hybridization-based rRNA depletion and poly(A)-based depletion step. For hybridization, cDNA oligonucleotides attach to complementary rRNA that is immobilized on magnetic beads; always ensure that the oligonucleotides are compatible with your organism(s) of interest. For this, we combine an equivalent volume of Ribo-Zero beads from both a Human/Mouse/Rat-specific and Gram-negative bacteria-specific Ribo-Zero^TM^ rRNA Removal Kit (Epicenter), enabling simultaneous reduction of both host and bacterial rRNA. It is important to note that this method will not enrich immature mRNAs or non-coding RNAs; specific target enrichment techniques that are outside the scope of this protocol should be considered if these are of experimental interest. Aliquots of rRNA-reduced samples may be then subjected to poly(A) depletion to further enrich host mRNA transcripts and separate mRNA from rRNA. Poly(A)-depleted and rRNA-depleted eluates can also be further purified before being combined for library construction. As a guide, 1 μg of input RNA will yield <80 ng of rRNA-depleted RNA. The remaining RNA is concentrated and purified with a RNA Clean & Concentrator^TM^-5 kit (Zymo Research).

### Total rRNA-depleted RNA integrity and quantification

Prior to submission for sequencing, the RNA integrity, purity, and concentration should be assessed, however dual RNA-Seq experiments add additional complexity to the traditional methods. Given that rRNA has been depleted, it is not possible to properly visualize 28S and 18S rRNA banding patterns following agarose gel electrophoresis to estimate RNA integrity. However, low-molecular weight smearing after running a small RNA sample from dual RNA-Seq experiments on a gel is an early, yet imperfect indication of possible RNA degradation. Spectrophotometric analysis, usually with a Nanodrop instrument, is useful for estimating RNA concentration and purity where readings are taken at a wavelength of 260 nm and the ratio of *A*_260_/*A*_280_ readings are measured. As a guide, an *A*_260_ of 1.0 is equivalent to about 40 μg/mL of RNA. The *A*_260_/*A*_280_ ratio is a measurement of protein contamination in the sample, where pure RNA should have a ratio of ~2.0. Note, however, that this method can suffer from low accuracy and biases introduced by protein contamination (Baelde et al., 2001).

A Bioanalyzer (Agilent) is usually regarded as the gold standard for estimating RNA quality by calculating an RNA integrity number (RIN), an estimation of RNA degradation which ranges from 1 to 10, with 10 being the least-degraded and most quality RNA. However, the mixture of eukaryotic and prokaryotic RNA in a dual RNA-Seq sample confounds the RIN measurement and a reliable number cannot be obtained. Instead, the Bioanalyzer electropherogram is useful to detect the presence of carry-over rRNA and RNA degradation. For RNA quantification with the Bioanalyzer we generally use an RNA 6000 ladder as the reference and prefer to use RNA 6000 pico chips as the nanochips tend to suffer from reduced sensitivity. However, note that the accuracy of the assay will decrease if the maximum RNA concentration is exceeded for the chip. Please consult Table 3 when troubleshooting RNA quantification steps.

### Library preparation and sequencing

When preparing RNA for NGS, the broad steps include fragmentation and/or size selection, conversion to cDNA, adapter attachment, and quantification. NGS is a rapidly expanding field and there are currently a number of sequencing platforms currently available, including Illumina, SOLiD, Ion Torrent, Roche 454, Oxford Nanopore, and Pacific Biosciences, with specific advantages and library preparation kits associated with each. When deciding on a sequencing technology, the defining objective of the experiment should be considered; i.e., is the focus on all transcriptional events (coding, non-coding, anti-sense, and intergenic RNAs), just the coding mRNA transcripts, or just small RNAs (miRNA, snoRNA, snRNA; Head et al., 2014). Long-read sequencing offered by PacBio and Nanopore can often span full-length transcripts to provide a more accurate measurement of isoform structures, while Oxford Nanopore can directly sequence RNA, removing the requirement for cDNA conversion. However, more traditional technologies such as Illumina and Ion Torrent can generally provide more accuracy. This stage of the experiment is not specific to (dual) RNA-Seq and is usually outsourced to commercial enterprise or central sequencing facility, with each providing their own detailed instructions for sample preparation so the steps are outside the scope of this protocol. Nevertheless, here we provide some general guidelines based on our experience.

We generally use the TruSeq Sample Prep Kit for library preparation and sequencing by the Illumina platform. For this, the mRNA is chemically fragmented and primed with random hexamer primers. First-strand cDNA synthesis occurs using reverse transcriptase, followed by second strand cDNA synthesis using DNA polymerase I and RNase H. The cDNA is purified and end-repaired and 3′ adenylated. Adapters containing six nucleotide indexes are ligated to the double-stranded cDNA, which is purified with AMPure XT beads (Beckman Coulter) and enriched via polymerase chain reaction (PCR) amplification. Paired-end reads >50 nucleotides will promote increased fragment randomization; longer reads will enable greater coverage, reduced multi-mapping, and improved transcript identification (Garber et al., 2011); we recommend paired-end 100 bp reads.

### Bioinformatics analysis

The latter stages of a dual RNA-Seq experiment are the key statistical and bioinformatic analyses that underlie the inference of biological knowledge. The difficulties of dealing with both prokaryotic and eukaryotic transcriptional data extend to these steps and several strategies have been developed to ensure meaningful data can be produced. While a brief overview is provided here, the researcher is encouraged to consult the detailed bioinformatic pipeline that has been published previously (Marsh et al., 2017).

Raw sequences are usually provided from the sequencing facility in FASTQ format, which can initially be subjected to contamination detection with FastQ Screen (www.bioinformatics.babraham.ac.uk/projects/fastq_screen/) to ensure that the majority of reads are derived from the two organisms of interest. This is followed by an assessment of read quality with FASTQC (www.bioinformatics.babraham.ac.uk/projects/fastqc/) and the use of Trimmomatic (Bolger et al., 2014) to remove low-quality reads and sequencing adapters. HISAT2 (Kim et al., 2015) is used for mapping and alignment, where total host-bacteria reads are mapped to the host reference genome, while unmapped (bacterial) reads are subsequently mapped to the bacterial reference genome with Bowtie2 (Langmead et al., 2009). We use HTSeq (Anders et al., 2015) or featureCounts (Liao et al., 2014) for read counting, where aligned sequences are quantified as an exon, transcript, gene etc., which results in a separate count matrix for host and pathogen reads, each consisting of genes (rows) and samples (columns). A key step prior to statistical analysis is data normalization. There are several normalization strategies available; we prefer the trimmed mean of *M*-values (TMM) method for both host and bacterial reads, which provides between-sample normalization while correcting for variations in sequencing depth and sample variation (Robinson and Oshlack, 2010).

For the actual data analysis, there are two distinct strategies for representing host and bacterial counts. For the host, genes that are differentially expressed between infected and non-infected controls are identified using packages such as edgeR, Kallisto, Limma, DESeq2, or Cufflinks, where we prefer the Limma package for dual RNA-Seq due to its ability to manage sample variation between conditions and time-points (Robinson et al., 2010; Trapnell et al., 2012; Love et al., 2014; Ritchie et al., 2015; Bray et al., 2016). This results in a list of DEGs, which are subjected to a false discovery rate (FDR) cutoff of <0.05 and a log fold-change (LFC) cutoff of at least 2-fold upregulation or downregulation. For the bacterial data, counts are subjected to within-sample normalization and reported as transcripts-per-million (TPM) which incorporates gene length information to provide a measurement of relative transcript abundance.

## Materials and equipment

### Reagents

Mammalian cells and associated media **CRITICAL** All experiments that use human or animal tissues must comply with governmental and institutional guidelines and regulations.

Bacterial species of interest **CAUTION** Many bacteria are human pathogens that pose a risk of infection. All work with this organism should be conducted in a class II biosafety cabinet while wearing appropriate personal protective equipment (PPE).

Ethanol (Sigma-Aldrich, cat. no. E7023) **CAUTION** Highly flammable. Causes skin and serious eye irritation. Handle using appropriate safety equipment.

Isopropanol (Sigma-Aldrich, cat. no. I9516)

Poly(A)Purist Mag Purification Kit (Thermo Fisher, cat. no. AM1922)

TURBO DNA-*free*^TM^ kit (Thermo Fisher, cat. no. AM1907)

DEPC-treated water (Thermo Fisher, cat. no. AM9915G)

MasterPure^TM^ Complete DNA and RNA Purification Kit (Epicenter, cat. no. MC85200)

RNA Clean & Concentrator^TM^-5 (Zymo Research, cat. no. R1015)

Ribo-Zero^TM^ rRNA Removal Kit (Human/Mouse/Rat) (Epicenter, cat. no. RZH1046)

Ribo-Zero^TM^ rRNA Removal Kit (Gram-negative bacteria) (Epicenter, cat. no. RZNB1056)

Sonicator S-4000 (Misonix, cat. no. S4000) (for use when lysing Gram positive bacteria)

TaqMan® Gene Expression Assay (Thermo Fisher, cat. no. 4453320)

High-Capacity cDNA Reverse Transcription Kit (Thermo Fisher, cat. no. 4368814)

Beta actin probe (Thermo Fisher, cat. no. Hs99999903_m1)

Mitochondrially encoded ATP synthase 6 probe (Thermo Fisher, cat. no. Hs02596862_g1)

Eukaryotic 18S rRNA probe (Thermo Fisher, cat. no. Hs99999901_s1)

### Equipment

MicroAmp® optical 96-well reaction plates (Thermo Fisher, cat. no. 4306737)

MicroAmp® optical adhesive film (Thermo Fisher, cat. no. 4311971)

MicroAmp® optical film compression pad (Thermo Fisher, cat. no. 4312639)

Flask rocker (Grant Instruments, PS-M3D)

DNase and RNase-free 1.5 mL microcentrifuge tubes (Sarstedt, cat. no. 72.692.210)

Refrigerated microcentrifuge (Beckman Coulter, 20R)

Centrifuge (Beckman Coulter, X-12R)

Vortex (Scientific Industries, G-560E)

Heating block (x2) (Thermo Fisher, cat. no. 2001Q)

Ice

Ice bucket

Timer

Real-time qPCR machine (Applied Biosystems, 7900HT)

Temperature cycler (Bio-Rad, C1000)

Incubator, 37°C, 5% CO_2_ (Sanyo, MCO-19AIC)

Bioanalyzer

RNA 6000 Pico Kit (Agilent, cat. no. 5067-1513)

6-well plates (Thermo Scientific, cat. no. 140675)

Cell scrapers (Sarstedt, cat. no. 83.1830)

15 mL centrifuge tubes (Thermo Scientific, cat. no. 339652)

### Reagent setup

**70% ethanol** Combine 350 mL ethanol with 150 mL nuclease free water. Store at room temperature for up to 6 months.

**Ribo-Zero^TM^ rRNA Removal Kit** The Ribo-Zero kits are composed of two parts: the magnetic core kit and the rRNA removal reagents. Store the magnetic core kits at 4°C and Mouse/Human/Rat and Gram-negative bacteria rRNA removal reagents at −80°C.

**RNA Clean & Concentrator^TM^-5** Add 48 mL of 100% ethanol to the 12 mL RNA Wash Buffer before use.

## Procedures

### Seeding and infection

1. Seed × 10^5^ mammalian host cells per well in all wells of a six-well plate. Ensure there are two plates per time-point (one infected plate and one non-infected control plate). Incubate plates overnight at 37°C, 5% CO_2_.

Following seeding, sit plates on a bench at room temperature for 15 min to allow cells to settle, ensuring an even distribution of cells.

The next day, the cells should be evenly distributed throughout the well.

2. The next day, infect all plates (except mock-infected control plates) at an MOI of 1.5 to ensure that 100% of the host cells will be infected.

Rock plates for 30 mins on shaker.

3. Centrifuge plates at 500 × g for 30 min at room temperature. Incubate plates at 37°C, 5% CO_2_, according to time-point.

Centrifugation is important to synchronize the infections and time-points and ensure an MOI of 1.5.

4. Wash cells twice with DPBS and overlay with warm, fresh media. Incubate at 37°C, 5% CO_2_.

### Harvesting cells (30 min)

5. At each time-point, wash cells twice with ~3 mL DPBS and add 1 mL DPBS to each well. Harvest cells with a cell scraper and dispense solution into a 15 mL centrifuge tube.

Ensure all cells are collected from each well. It may be beneficial to add a second 1 mL of DPBS to collect any remaining cells.

Store tubes at −80°C until all time-points are complete. Cells can be stored for up to 6 weeks at −80°C.

## Pause point

### Cell lysis (45 min)

6. Remove centrifuge tubes from −80°C freezer and thaw at room temperature.
7. Pre-set heating block to 65°C.
8. Add 1 μL of 50 μg/mL Proteinase K (MasterPure^TM^ Complete DNA and RNA Purification Kit; Epicenter) to 300 μL of Tissue and Cell Lysis Buffer (MasterPure^TM^ Complete DNA and RNA Purification Kit; Epicenter) for each sample.
9. Pellet cells by centrifugation at 5,000 × g for 30 min. Discard the supernatant, leaving ~25 μL of liquid.
10. Vortex for 10 s to resuspend the pellet.
11. Add 300 μL of Tissue and Cell Lysis Solution (containing Proteinase K) to each 25 μL sample and mix thoroughly by vortexing.
12. **For systems containing Gram positive bacteria**:

Set sonicator to an amplitude setting of 10 and perform six treatments of 30 s each to disrupt the cells. Place tubes on ice for 30 s between each treatment.

Incomplete sonication can lead to a reduction in bacterial RNA yield.

13. **For all systems**:

Incubate tubes in a heating block at 65°C for 15 min, vortexing briefly every 5 min.

The solution should become cloudy as cells are lysed and proteins are digested.

14. Place samples on ice for 3–5 min.

### Total nucleic acid precipitation (1 h)

15. Add 175 μL of MPC Protein Precipitation Reagent (MasterPure^TM^ RNA Purification Kit; Epicenter) to each 300 μL of lysed sample and vortex vigorously for 10 s.
16. Pellet the debris by centrifugation at 10,000 × g for 10 min at 4°C.

You should see a clear pellet of cellular debris. If not, vortex briefly, incubate at room temperature for 10 mins and centrifuge again.

17. Transfer the supernatant (containing total nucleic acid) to a clean 1.5 mL microcentrifuge tube and discard the pellet.
18. Add 500 μL of isopropanol to the recovered supernatant and invert the tube 30–40 times. Do not vortex.

Isopropanol will desalt and precipitate the nucleic acid. It may be beneficial to mark the side of the tube prior to centrifugation as pellets from isopropanol treatment have a glassy appearance that may be difficult to see.

19. Pellet the total nucleic acid by centrifugation at 10,000 × g for 10 min at 4°C.
20. Carefully pour off the isopropanol without dislodging the pellet.

Pellets from isopropanol treatment are loosely attached and care should be taken when pouring.

21. Rinse the pellet twice with 70% ethanol, being careful not to dislodge the pellet. Centrifuge briefly if pellet is dislodged.

Ethanol is more volatile than isopropanol and will make the DNA easier to dissolve. Perform at room temperature.

22. Remove residual ethanol with a pipette and resuspend the pellet in 35 μL of TE Buffer (MasterPure^TM^ RNA Purification Kit; Epicenter). Samples can be stored in TE Buffer overnight at 4°C.

## Pause point

### DNA digestion (45 min)

23. Pre-set heating block to 37°C.
24. Add 4 μL of 10x TURBO Dnase Buffer (TURBO^TM^ DNA-*free*^TM^ Kit; Thermo Fisher) to each 35 μL sample.
25. Add 1 μL of TURBO^TM^ DNase to each sample. Gently flick the tubes to mix and briefly centrifuge to distribute liquid to the bottom of the tube.

Increase DNase volume to 2-3 μL if digesting a higher amount of DNA. Alternatively, add half the DNase to each reaction, incubate for 30 min, then add the remainder of the enzyme and incubate for another 30 min.

26. Incubate tubes in a heating block at 37°C for 30 min.
27. After incubation, add 8 μL of DNase Inactivation Solution (TURBO^TM^ DNA-*free*^TM^ Kit; Thermo Fisher) and incubate tubes at room temperature for 5 min. Mix occasionally by manually inverting.

Environments colder than 22°C can reduce the inactivation of TURBO^TM^ DNase. Move tubes to a heating block to control the temperature if necessary.

28. Centrifuge tubes at 10,000 × g for 1.5 min.
29. Transfer supernatant (containing the RNA) to a fresh 1.5 mL microcentrifuge tube.

The pellet should be visible; if not, vortex briefly and repeat centrifugation for 5 mins. Take care not to dislodge the pellet when pouring.

## Pause point

### Validation of DNA removal by RT-PCR (2 h)

30. Remove 5 μL from each RNA sample and place in a clean 1.5 mL microcentrifuge tube.
  Keep all samples and reactions on ice.
31. Add 95 μL of nuclease-free water (1:20 dilution).
32. Prepare a 2X RT master mix for each sample in triplicate. Include a non-template control and an RT- control.
10X RT buffer: 2 μL
25X dNTP mix (100 mM): 0.8 μL
10X RT random primers: 2 μL
RNase inhibitor: 1 μL
RNA-free dH_2_O: 3.2 μL
33. Prepare 1X reactions (20 μL total) by combing the 2X master mix with the following in individual wells of a 96-well reaction plate (Thermo Fisher):
Reaction 1: 10 μL sample + 2 μL RT + 1 μL dH_2_O
Reaction 2: 10 μL sample + 3 μL dH_2_O
Reaction 3: 11 μL dH_2_O + 2 μL RT
Gently pipette up and down to mix.
Seal the plate.
34. Briefly centrifuge plate to spin down the solution and eliminate any air bubbles.
35. Perform the reverse transcription reaction according to the following conditions:
25°C, 10 min
7°C, 120 min
85°C, 5 min

cDNA can be stored at −20°C. Create small aliquots to prevent degradation resulting from frequent freeze-thawing.

36. Run 20 μL of each reaction on a 1% agarose gel and examine under UV fluorescence.
  There should be large bands in the lane containing Reaction 1, indicative of cDNA. The lane containing Reaction 2 should be free of bands, otherwise contamination with gDNA has occurred. The lane containing Reaction 3 should also be free of bands. See Table 3 for troubleshooting options.

### Confirm presence of RNA by RT-PCR

37. Make a 1:20 dilution of cDNA, prepared above.
38. Prepare enough RT-PCR master mix to assay each sample in triplicate, as well as controls.
20x TaqMan® Gene Expression Assay: 1 μL
2x TaqMan® Gene Expression Master Mix: 10 μL
RNase-free water: 5 μL
39. Cap the tube and invert the tube several times to mix the reaction components. Pulse vortex.
40. Aliquot 15 μL of master mix into individual wells of a 96-well reaction plate (Thermo Fisher). Include a triplicate reaction for each sample and non-template controls.
41. Add 4 μL of each diluted template to appropriate wells and gently tap plate on benchtop to distribute contents to the base of the well.
42. Place adhesive film (Thermo Fisher) over the plate and seal with compression pad (Thermo Fisher).

If any bubbles are visible in the wells or liquid is present on the sides of the wells, centrifuge plate at 500 × g for 2 min. Do not touch the film with ungloved hands at any point.

43. Place plate in RT-PCR machine and run assay according to the following cycling conditions:
Hold: 95°C, 10 min
Cycle (40x): 95°C, 15 s
60°C, 1 min.

Amplification curves should be present for all targets, representing the accumulation of product throughout the RT-PCR experiment. Ensure that the amplification signal is at least ten times the baseline standard deviation to indicate amplification over noise. See Table 3 for additional guidelines.

## Pause point

### rRNA depletion (90 min)

44. Pre-set one heating block to 68°C and one at 50°C.
45. Remove Ribo-Zero^TM^ rRNA Removal Magnetic Core Kit from 4°C and allow to warm to room temperature. Remove Human/Mouse/Rat and Gram-negative bacteria components of the Ribo-Zero^TM^ rRNA Removal kits from −80°C and thaw on ice.

Do not place the Ribo-Zero^TM^ Magnetic Core Kit on ice as this can damage them and reduce the efficiency of the procedure.

46. Vigorously mix magnetic beads (Ribo-Zero^TM^ rRNA Removal kit; Illumina) for 20 s by vortexing.
47. Carefully pipette 65 μL of magnetic beads into 2 mL microsphere wash tubes (Ribo-Zero^TM^ rRNA Removal kit; Illumina); two tubes per sample.

Dispense magnetic beads slowly to avoid introducing air bubbles. Store unused beads at 4°C. Do not place the magnetic beads on ice.

48. Open tube cap and place on magnetic stand for 2 mins.

At this stage the liquid should become clear.

49. Centrifuge microspheres at 12,000 × g for 3 min. Carefully remove supernatant without dislodging the pellet.

**HAZARD:** The supernatant contains sodium azide.

50. Wash the microsphere wash tubes by adding 130 μL of microsphere wash solution (Ribo-Zero^TM^ rRNA Removal kit; Illumina) to each tube. Vortex vigorously.
51. Centrifuge microsphere wash tubes at 12,000 × g for 3 min. Carefully remove supernatant without dislodging the pellet.
52. Add 65 μL of microsphere resuspension solution (Ribo-Zero^TM^ rRNA Removal kit; Illumina) to each tube and vortex vigorously until a homogenous suspension is produced.
53. Add 1 μL of RiboGuard RNase inhibitor (Ribo-Zero^TM^ rRNA Removal kit; Illumina) to each tube. Mix by vortexing for 10 s and set aside (at room temperature).

Avoid creating air bubbles when adding RNase inhibitor.

54. Treat two aliquots of each sample with Ribo-Zero rRNA removal solution (Ribo-Zero^TM^ rRNA Removal kit; Illumina) according to the following preparation (two removal preps per sample):
RNase-free water (Ribo-Zero^TM^ rRNA Removal Kit): 1 μL
Ribo-Zero Reaction Buffer: 4 μL
RNA sample: 25 μL
Ribo-Zero rRNA Removal Solution (Gram negative bacteria kit): 5 μL
Ribo-Zero rRNA Removal Solution (Human/Mouse/Rat kit): 5 μL

Fully mix the samples by pipette-mixing 10-15 times.

The beads may clump at this stage due to the biotin on the probes binding to multiple beads. If so, vortex for at least 10 s.

55. Incubate at 68°C for 10 min in heating block. Return the Ribo-Zero reaction buffer to −80°C.
56. Remove the microsphere wash tubes from the heating block and incubate at room temperature for 15 min.
57. Vortex the microsphere wash tubes at medium speed for 20 s to ensure a homogenous slurry.
58. Pipette hybridized RNA sample to the resuspended microsphere wash tubes, pipette-mixing 10–15 times to mix. Immediately vortex the microsphere wash tubes at medium speed for 5 s.

The washed magnetic beads must be at room temperature for use in this step. The order of the addition (hybridized RNA *to* the magnetic beads) is critical for rRNA removal efficiency.

59. Incubate microsphere wash tubes at room temperature for 10 min. Vortex at medium speed for 5 s, every 3–4 min.
60. Following incubation, mix samples again by vortexing at medium speed for 5 s.
61. Incubate samples in heating block at 50°C for 10 min.
62. Transfer the RNA-microsphere suspension to a Microsphere Removal Unit (Ribo-Zero^TM^ rRNA Removal kit; Illumina) and centrifuge at 12,000 × g for 1 min at room temperature. Save the eluate and discard the removal unit.

At this stage, the eluate should be ~100 μL.

Probe and rRNA contamination can occur if magnetic beads are allowed to carry over.

## Pause point

### Purification of rRNA-depleted samples (45 min)

63. Add 2 volumes of RNA Binding Buffer (RNA Clean & Concentrator^TM^-5; Zymo Research) to each volume of RNA sample and mix well.

The minimum recommended sample volume for use with this kit is 50 μL.

64. Add 1 volume of 100% ethanol to the mixture and mix well.
65. Transfer the mixture to a Zymo-Spin IC column (RNA Clean & Concentrator^TM^-5; Zymo Research) in a collection tube and centrifuge at 12,000 × g for 1 min. Discard the flow-through.
66. Combine two reactions of the same sample to one column and spin multiple times until the entire mixture passes through the column. The column capacity is 5 μg of RNA.
67. Add 400 μL of RNA Prep Buffer (RNA Clean & Concentrator^TM^-5; Zymo Research) to the column and centrifuge at 12,000 × g for 1 min. Discard the flow-through.
68. Add 800 μL of RNA Wash Buffer (RNA Clean & Concentrator^TM^-5; Zymo Research) to the column and centrifuge at 12,000 × g for 1 min. Discard the flow-through.
69. Add 400 μL of RNA wash buffer to the column and centrifuge at 12,000 × g for 1 min. Discard the flow-through.
70. Centrifuge the column in an emptied collection tube at 12,000 × g for 2 min. Carefully remove the column from the collection tube and transfer to a new RNase-free microcentrifuge tube.
71. Add 20 μL of DNase/RNase-free water directly to one column matrix and let stand for 10 min at room temperature. Centrifuge at 10,000 × g for 30 s.

Repeat elution.
The eluted RNA can be used immediately or stored at −80°C.

## Pause point

### Check RNA quality and quantity

72. Make a 1:20 dilution of eluted RNA.
73. Run on a 2% agarose gel with an appropriate molecular weight marker.

Large, clear bands are likely to be rRNA, indicating that rRNA depletion was not successful. Look for low molecular weight smearing which will indicate RNA degradation. High(er) molecular weight smearing (> ~400 bp) will be the eluted RNA and can be expected. See Table 3 for troubleshooting options.

74. Measure the quality and quantity of eluted RNA by assaying a 1 μL on a Nanodrop instrument. Observe absorbance readings at 260 nm, as well as the *A*_260_/*A*_280_ ratio.

The RNA concentration should be sufficient for the sequencing requirements (check the sample submission guidelines for the sequencing technology that will be used). An *A*_260_ of 1.0 is equivalent to about 40 μg/mL of RNA. The *A*_260_/*A*_280_ ratio is a measurement of protein contamination in the sample, where pure RNA should have a ratio of ~2.0.

75. Measure the quantity and integrity of RNA on a Bioanalyzer instrument. Consult the manufacturer's guidelines for the assay: http://www.genomics.agilent.com/en/Bioanalyzer-System/2100-Bioanalyzer-Instruments/?cid=AG-PT-106

If carryover rRNA is present, the electropherogram will produce two distinct peaks corresponding to 18S and 28S eukaryotic rRNA (and possibly 16S and 23S prokaryotic rRNA, but these cannot be differentiated from the eukaryotic rRNA peaks), indicating that rRNA-depletion was not successful. Degraded RNA can be identified by any peaks appearing at the lower part of the electropherogram, which highlights a distribution shift toward smaller RNA fragments. See Table 3 for additional troubleshooting steps.

## Pause point

### Library preparation and sequencing

The method of library preparation is dependent on the sequencing platform used and thus is outside the scope of this protocol. Commercial sequencing enterprises and sequencing centers will provide detailed guidelines on the library preparation and sample submission guidelines that begin with the isolation of pure mRNA as described above. Following sequencing, the user will be provided with a series of raw FASTQ sequence files that serve as the input for the subsequent computational data analysis.

## Author contributions

JM, MH designed the protocol. JM, MH, GM wrote the manuscript.

### Conflict of interest statement

The authors declare that the research was conducted in the absence of any commercial or financial relationships that could be construed as a potential conflict of interest.
